# Evaluation of Plaque Stability of Advanced Atherosclerotic Lesions in Apo E-Deficient Mice after Treatment with the Oral Factor Xa Inhibitor Rivaroxaban

**DOI:** 10.1155/2011/432080

**Published:** 2011-06-07

**Authors:** Qianxing Zhou, Florian Bea, Michael Preusch, Hongjie Wang, Berend Isermann, Khurrum Shahzad, Hugo A. Katus, Erwin Blessing

**Affiliations:** ^1^Department of Internal Medicine III, University of Heidelberg, 69120 Heidelberg, Germany; ^2^Department of Cardiology, Tongji Hospital, Huazhong University of Science and Technology, Wuhan 430030, China; ^3^Department of Internal Medicine I, University of Heidelberg, 69120 Heidelberg, Germany

## Abstract

*Aim*. Thrombin not only plays a central role in thrombus formation and platelet activation, but also in induction of inflammatory processes. Activated factor X (FXa) is traditionally known as an important player in the coagulation cascade responsible for thrombin generation. We assessed the hypothesis that rivaroxaban, a direct FXa inhibitor, attenuates plaque progression and promotes stability of advanced atherosclerotic lesions in an *in vivo* model. *Methods and Results*. Rivaroxaban (1 or 5 mg/kg body weight/day) or standard chow diet was administered for 26 weeks to apolipoprotein E-deficient mice (*n* = 20 per group) with already established atherosclerotic lesions. There was a nonsignificant reduction of lesion progression in the high-concentration group, compared to control mice. FXa inhibition with 5 mg Rivaroxaban/kg/day resulted in increased thickness of the protective fibrous caps (12.3 ± 3.8 *μ*m versus 10.1 ± 2.7 *μ*m; *P* < .05), as well as in fewer medial erosions and fewer lateral xanthomas, indicating plaque stabilizing properties. Real time-PCR from thoracic aortas revealed that rivaroxaban (5 mg/kg/day) treatment reduced mRNA expression of inflammatory mediators, such of IL-6, TNF-*α*, MCP-1, and Egr-1 (*P* < .05). *Conclusions*. Chronic administration of rivaroxaban does not affect lesion progression but downregulates expression of inflammatory mediators and promotes lesion stability in apolipoprotein E-deficient mice.

## 1. Introduction

Rivaroxaban (Bayer AG, Germany) is a novel oral direct factor Xa (FXa) inhibitor not only of free FXa, but also of prothrombinase complex and clot bound FXa activity. It displays potent antithrombotic effects in a variety of *in vivo* venous and arterial thrombosis models [1]. 

In 2008, rivaroxaban received approval in the European Union and in Canada for the prevention of VTE in adult patients undergoing elective total hip or knee replacement surgery. Two major clinical studies evaluated rivaroxaban in patients with atrial fibrillation [2] and in the treatment of venous thromboembolism [3]. Besides its classical role in the prevention or treatment of thromboembolic diseases, rivaroxaban was also evaluated in a phase II clinical trial in patients stabilized after acute coronary syndromes (ACS). The risk of clinically significant bleeding was increased in a dose-dependent manner, as compared with placebo. Factor Xa inhibition was associated with a trend reduction of the primary efficacy endpoint of death, myocardial infarction, stroke, or severe recurrent ischaemia requiring revascularisation. Regarding the main secondary efficacy endpoint, rivaroxaban significantly reduced the rate of death, myocardial infarction, or stroke [4]. The phase III study Atlas-ACS-TIMI 51 in patients after ACS is ongoing.

Atherosclerosis is a progressive, inflammatory disease characterized by the accumulation of lipids and fibrous elements in the arteries. Although advanced lesions can grow sufficiently large to block blood flow, the most important clinical complication is an acute occlusion due to thrombus formation, resulting in myocardial infarction or stroke. Often, thrombus formation is associated with rupture or erosion of unstable atherosclerotic lesion, as the prothrombotic content of necrotic cores get exposed to circulating thrombocytes [5, 6].

The intrinsic and the extrinsic pathway of the coagulation cascade converge at the activation of factor X to Xa. Active factor Xa hydrolyzes and activates prothrombin to thrombin. Increasing evidence showed that coagulation factors such as thrombin participate in atherosclerotic heart disease in ways that do not directly involve thrombus formation such as signalling through protease-activated receptors [7]. Previous experiments in our laboratory demonstrated that ximelagatran, a direct thrombin inhibitor, reduced lesion progression and promoted plaque stability in apolipoprotein E-deficient mice [8]. Data on the vascular effects of new generation direct FXa inhibitors are very limited.

This prompted us to investigate whether administration of the direct factor Xa inhibitor rivaroxaban attenuates progression and promotes stability of advanced atherosclerotic lesions in hyperlipidemic apolipoprotein E-deficient mice.

## 2. Materials and Methods

### 2.1. Animals and Drug Treatment

Sixty 26-week-old female apoliprotein E-deficient mice (Charles River Laboratories, Wilmington, USA, strain name B6.129P2Apoe^tm1Unc^/Crl) with already established advanced atherosclerotic lesions in the innominate artery were kept within the animal care facility of the University of Heidelberg. Sixty mice were randomized to 3 groups (20 mice per group): one group received standard chow diet (control group), one group received chow diet supplemented with 1 mg rivaroxaban/kg bodyweight/day (low-concentration group), and one group a chow diet, supplemented with 5 mg rivaroxaban/kg bodyweight/day (high-concentration group) for 26 weeks. The housing and care of animals and all the procedures done in the study were performed in accordance with the guidelines and regulations of the local Animal Care Committee of the University of Heidelberg. The investigation conforms to the Guide for the Care and Use of Laboratory Animals published by the US National Institutes of Health (NIH publication no. 85–23, revised 1996).

### 2.2. Animal Sacrifice and Preparation of Plasma and Tissue

Mice were euthanized at 52 weeks without fasting before anaesthesia. Mice were heavily sedated (Xylazin and Ketamin), blood was collected in citrated (5 mice per group) or heparin (5 mice per group) syringes from the inferior vena cava and saved in citrated or heparin vials. Blood samples were centrifuged at 3000 rpm for 10 min at approximately 4°C to obtain plasma, which was stored at −20°C until analysis. Mice were then perfused with 10 mL phosphate buffered saline at physiological pressure via left ventricle, and thoracic aortas were ligated distal of the left subclavian artery and removed for subsequent analysis of candidate genes by RT-PCR or for transcription factor analysis by gel shift assays. Then, mice were perfused with 4% buffered formalin, at physiological pressure via the left ventricle. Finally, innominate arteries were embedded in paraffin and serially sectioned (5 *μ*m). Every fifth section was stained with Movat's staining. 

### 2.3. Determination of Plasma Lipid Levels and Rivaroxaban Concentration

Plasma concentrations of total cholesterol, HDL and LDL cholesterol, and triglycerides were determined enzymatically in heparinized plasma with an automatic analyzer ADVIA 2400 Chemistry Systems (Bayer AG, Germany). Total cholesterol and triglycerides were measured using the COD-PAP and GPO-PAP method (cholesterol oxidase/glycerol phosphate oxidase coupled to phenol and 4-aminophenazone) and HDL and LDL cholesterol were measured using commercial available kits (Bayer AG, Germany). Rivaroxaban concentrations were determined in citrated plasma by Bayer AG, Germany.

### 2.4. Evaluation of Plaque Composition and Lesion Size

Two independent investigators who were blinded to the study protocol evaluated each section for characteristic features of plaque instability. These included thickness of the fibrous cap (thin fibrous cap was defined as 3 or fewer cell layers), size of the necrotic core (a large necrotic core was defined as occupying more than 1/3 of the total lesion area), intraplaque hemorrhage (defined as the presence of red blood cells independent of microvessels), calcification (defined on the basis of positive staining with the van Kossa stain), presence of cholesterol crystals, medial erosion (defined as infiltration of plaque tissue into the media), and lateral xanthomas (defined as the presence of aggregates of macrophage-derived foam cells situated on the lateral margins of the plaques). These were recorded as binary outcomes and the frequency for each group was determined. 

Each cross-sectional area of each plaque was analyzed using computer-assisted morphometry (Image Pro, Media Cybernetics, Silver Spring, USA). Maximum lesion area, maximum lesion thickness, maximum percent stenosis, maximum area of the necrotic core and minimal thickness of the fibrous cap and of the media per animal were identified and used for subsequent statistical comparison between the groups. 

### 2.5. Immunohistochemistry

Tissue sections of the innominate artery adjacent to the sites of maximum lesion area were dewaxed and rehydrated. Endogenous peroxidase activity was inhibited by incubation with peroxoblock (Invitrogen, Karlsruhe, Germany). Detection of macrophages was performed by using monoclonal rat anti-mouse antibody (anti-Mac-2, Burlington, Canada), expression of smooth muscle alpha actin using a specific rabbit antibody (Dianova GmbH, Hamburg, Germany) according to the manufacturer's protocols. Anti-Mac-2 and actin were incubated for one hour at room temperature. Sections were then incubated with biotinylated secondary antibodies, rinsed 3x with PBS and incubated for 10 min with streptavidin at room temperature. AEC-chromogen substrate (Zytomed Systems GmbH, Berlin, Germany) was used for visualization. The extent of positive staining within the lesions was normalized to maximum lesion area using computer-assisted morphometry (Nikon GmbH, Düsseldorf, Germany).

### 2.6. Real-Time PCR Analysis

Total RNA from thoracic aortas was extracted and determined by absorbance at 260 nm. RNA was reverse transcribed with revert aid first strand synthesis kit (Fermentas, St. Leon-Rot, Germany). Real-time PCR was performed for each gene on a light cycler using hybridization probes and primers designed on the Universal Probe Library (Roche Diagnostics GmbH, Mannheim, Germany). All procedures were performed according to manufacturer's protocol. Relative levels of gene expression were calculated as previously reported [9].

### 2.7. Statistical Analysis

All data are expressed as mean ± S.E.M. Significant differences between means in plasma lipid levels, serum rivaroxaban levels were determined with one-factor ANOVA. Analysis of plaque morphometry and areas of positive immunohistochemistry staining were determined by using one-factor ANOVA. For evaluation of plaque morphology, groups were compared using *χ*² test. *P* values ≤.05 were considered statistically significant.

## 3. Results

### 3.1. Body Weights, Lipid Levels, and Rivaroxaban Plasma Concentrations

One mouse out of the control group died during the study. All other mice remained healthy. In particular, no apparent bleeding was observed. At the time of sacrifice, mice treated by the high dose of rivaroxaban showed slightly, yet significant lower body weights compared to the control group (Table 1).

There were no significant differences in total cholesterol, LDL-cholesterol, HDL-cholesterol, and total triglycerides levels among the groups (Table 1). Therefore, the observed effects in the present study are not related to any lipid-modifying effects of rivaroxaban. Plasma levels of rivaroxaban were 24.2 ± 9.3 ng/mL (average ± SD) in the high-concentration group and 6.2 ± 1.5 ng/mL (average ± SD) in the low-concentration group (*n* = 5 in each group) (Table 1).

### 3.2. Lesion Progression and Plaque Composition

**Figure 1:**
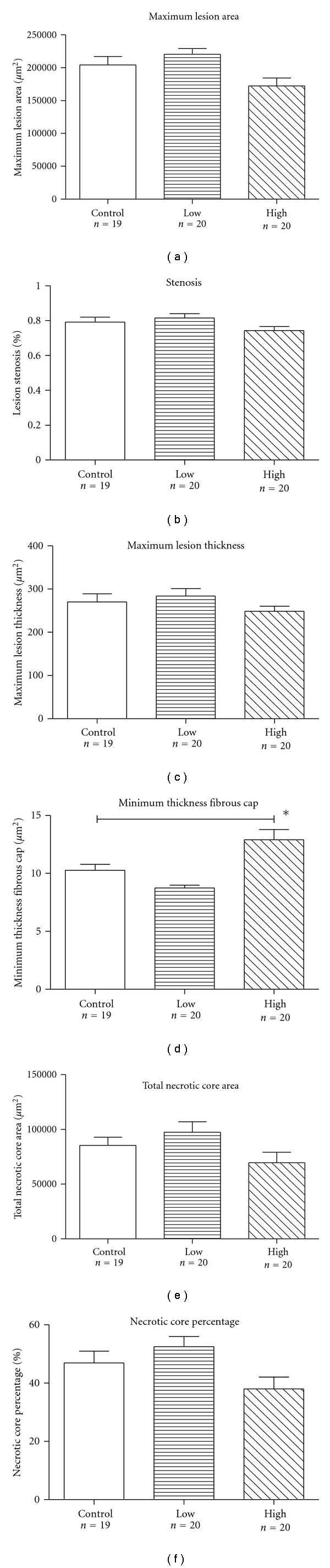
*Morphometric analysis data*. Administration of rivaroxaban in the chow diet over 26 weeks did not significantly reduce maximum lesion area (a), percentage of stenosis (b), lesion thickness (c), necrotic core area (e), or percentage of the necrotic core area in relation to total lesion area (f). Thickness of the protective fibrous cap was significantly larger in mice, receiving the high-concentration rivaroxaban, as compared to control mice ((d), *P* < .05). Data represent mean ± SEM. *P < .05.

Morphometric analysis of brachiocephalic arteries revealed that chronic administration of rivaroxaban in the chow diet over 26 weeks did not significantly alter progression of atherosclerotic plaques in apolipoprotein E-deficient mice (Figure 1). However, administration of the high concentration of rivaroxaban increased thickness of the protective fibrous caps (12.3 ± 3.8 *μ*m versus 10.1 ± 2.7 *μ*m of the control group; *P* < .05). Moreover, there was a significant reduction in the presence of medial erosions and lateral xanthomas in the rivaroxaban mice (5 mg/kg/day), as compared with the control animals (Table 2). Interestingly, frequency of medial erosions were also reduced in our previous experiments with the direct thrombin inhibitor melagatran [8]. Representative lesion morphology is shown in Figure 4.

**Figure 2:**
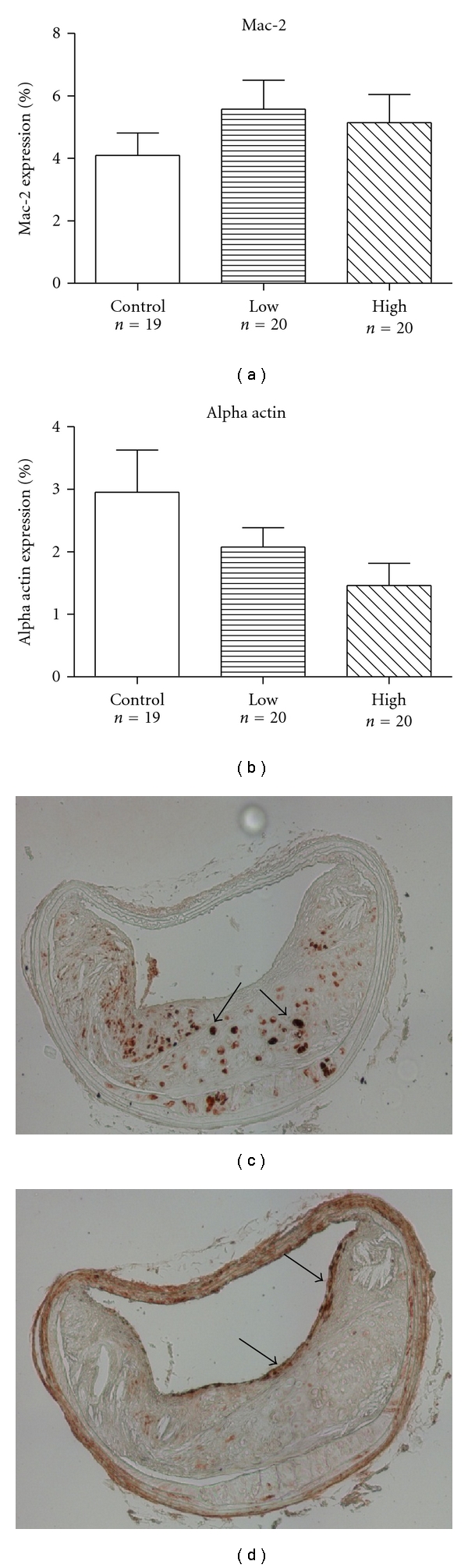
*Immunohistochemistry*. Immunohistochemistry staining with antibodies against Mac-2 (a, c), demonstrating presence of macrophages within an advanced lesion. Mac-2 was predominantely located within the necrotic cores (arrows) and did not significantly differ between the three experimental groups. Staining for alpha actin (b, d) was predominantely located within the fibrous cap (arrows) and showed a trend reduction (*P* = .06) in the high-concentration group, as compared to the control group. Both representative cross-sections (c, d) were from mice that received high-concentration of rivaroxaban (5 mg/kg/day). Error bars represent mean ± SEM.

Analysis of plaque composition by immunohistochemistry showed a nonsignificant decrease of staining against smooth muscle alpha actin (*P* = .06) in the high dose rivaroxaban group (Figure 2). Staining against smooth muscle alpha actin was predominantly located within the fibrous cap. There was no statistical significant difference in staining against Mac-2 between the three groups (Figure 2).

### 3.3. Expression of Inflammatory Cytokines in Aortic Tissue

**Figure 3:**
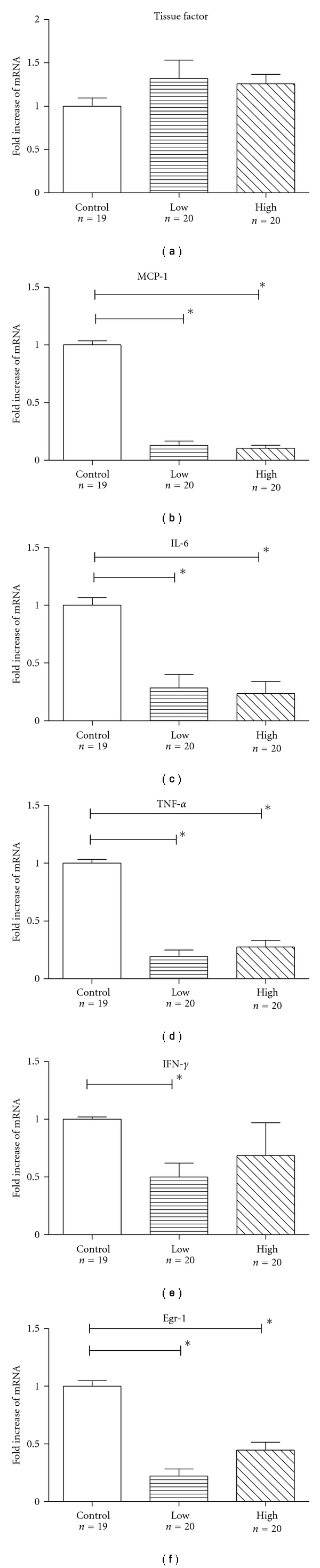
*RT-PCR of inflammatory mediators*. Chronic administration of both concentrations of rivaroxaban significantly decreased mRNA expression of MCP-1 (b), IL-6 (c), TNF-*α* (d), and Egr-1 (f) in aortic tissue of rivaroxaban-treated mice, as compared to the control group. Mice receiving the low (1 mg/kg/day), but not the high dose (5 mg/kg/day) of rivaroxaban, showed a significant reduction of mRNA expression of IFN-*γ* (e). There were no significant differences in respect of tissue factor expression between the three groups (a). Data represent fold increase of mRNA expression as compared to the control group. Data represent mean ± SEM. *P < .05.

**Figure 4:**
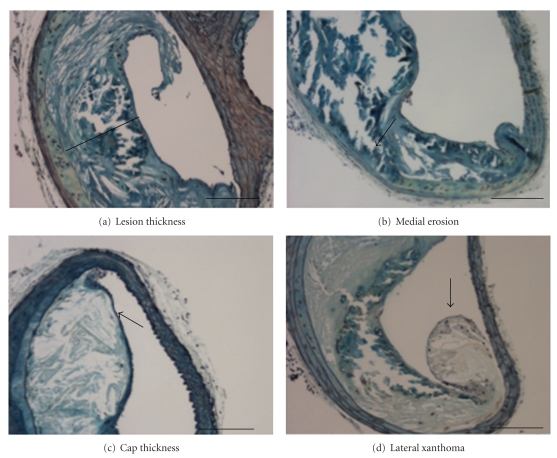
*Representative lesion morphology*. Representative brachiocephalic artery from a mouse after chronic administration of rivaroxaban (5 mg/kg/day). Movat's pentachrome staining shows features of an advanced but rather stable atherosclerotic lesion with a small necrotic cores and a thick protective fibrous cap (a). In contrast, a lesion from a mouse from the low-concentration group (1 mg/kg/day) shows a less stable lesion with a large necrotic core, partially eroding into the media (arrow, (b)). (c) shows a representative example of a control mouse, where the fibrous cap (arrow), separating the necrotic core from the lumen, is reduced to few cell layers. Lateral xanthomas (arrow), defined as the presence of aggregates of macrophage-derived foam cells, situated on the lateral margins of the plaques were observed significantly more frequently in control mice (d). Bar indicates 100 *μ*m. Data are presented as mean ± SEM.

Chronic administration of both concentrations of rivaroxaban significantly decreased mRNA expression of IL-6, TNF-*α*, MCP-1, and Egr-1 in aortic tissue of treated mice, as compared to the control group (Figure 3). Data in Figure 3 indicate the fold increase of mRNA expression of the aortic tissue in relation to that of control mice. Surprisingly, only mice receiving the low, but not the high dose of rivaroxaban, showed a significant reduction of mRNA expression of IFN-*γ*. There were no significant differences in respect of tissue factor expression between the three groups (Figure 3).

## 4. Discussion

The present study demonstrates that long-term administration of the oral direct factor Xa inhibitor rivaroxaban reduces expression of proinflammatory mediators, such as IL-6, TNF-*α*, MCP-1, and Egr-1 in aortic tissue of apolipoprotein E-deficient mice. Furthermore, rivaroxaban enhances thickness of the protective fibrous caps and reduces presence of medial erosions and lateral xanthomas, thus promoting lesion stability in this model of advanced atherosclerotic disease. However, the reduction of progression of lesion size did not achieve statistical significance. 

Factor Xa plays a central role in the coagulation cascade, linking the extrinsic and intrinsic pathways by catalyzing the conversion of prothrombin to thrombin on vascular cell surfaces [10]. Factor Xa elicits various and complex signalling events on a wide range of cell types, by activating protease-activated receptors-1 (PAR-1) and PAR-2. Factor Xa and thrombin share PAR-1, the main receptor for thrombin as a downstream cellular receptor [11]. PAR-2 expression is enhanced in human coronary atherosclerotic lesions [12]. Mediated through PAR-2, FXa acts as a powerful chemoattractant for fibroblasts and contributes to fibroblast differentiation into myofibroblasts [13]. Interest in Factor Xa signalling was ignited after the realization that PAR-2, rather than PAR-1, acts as a key player in the progression of a wide pattern of pathologies at the fibroproliferative interface [13–16]. 

Atherosclerosis is not only related to the formation and progression of atherosclerotic plaques, but also considered a systemic inflammatory disease, which is modulated by genetic and environmental risk factors. Sustained inflammation can lead to plaque rupture and thrombus formation with subsequent ischemia and myocardial infarction [5, 6]. In recent studies, Factor Xa has been found to trigger acute inflammatory responses *in vivo *[17–19] and *in vitro *[20–22]. In endothelial cells, Factor Xa leads to the activation of nuclear factor *κ*B (NF-*κ*B), the release of IL-6, IL-8, and monocyte chemotactic protein-1 (MCP-1), which contributes to leukocyte recruitment [20–22]. Most of these responses are mediated via PAR2 activation although some studies showed minor involvement of PAR1 [21, 23].

Coagulation is closely united with inflammatory signalling pathways [24, 25]. Several studies have shown that anticoagulant treatment not only diminishes activation of coagulation but also inhibits inflammation, indicating the extensive interplay between these processes. However, effects of rivaroxaban on lesion composition were, at least in part, unrelated to its antithrombotic activity. The expression of tissue factor, a potent activator of the extrinsic coagulation cascade, was not different in thoracic aortas from rivaroxaban treated compared with control mice. Significant thrombus formation was neither observed in the present study, nor in our previous studies of advanced atherosclerotic lesions in the same model, possibly due to a high fibrinolytic activity in mice [26].

Rivaroxaban is a small synthetic molecule able to inhibit free Factor Xa clot-bound and prothrombinase-bound Factor Xa. Unlike indirect Factor Xa inhibitors, the effect of rivaroxaban does not require the presence of antithrombin [27].

Factor Xa is also known to promote mitogenesis of serveral cell lines, including vascular smooth muscle cells (VSMC) [28–30]. Several studies demonstrated that inhibition of Factor Xa reduces VSMC proliferation [31], and restenosis after balloon angioplasty [31–35]. At the molecular level, wound healing and fibrosis share similar mechanisms, including fibroblast migration, proliferation and subsequent differentiation into myofibroblasts that typically express *α*-SM actin [36]. In renal interstitial fibrosis, there is a correlation between *α*-SM actin and PAR-2 expression [37]. In the present study, there was also a trend towards reduced staining against *α*-smooth muscle actin in lesions of rivaroxaban treated mice, suggesting a potential effect of rivaroxaban reducing restenosis after ballon angioplasty, through its antiproliferative properties.

Interestingly, in contrast to our previous observations with simvastatin [38], frequency of intraplaque haemorrhage was not reduced by administration of rivaroxaban. It is possible that in regard to plaque haemorrhage, plaque-stabilizing effects of Factor Xa inhibition were counteracted by its anticoagulatory properties, resulting in comparable patterns of erythrocyte deposition in otherwise more stable lesions.

Plasma levels of rivaroxaban were rather low in our study. Bioavailability of rivaroxaban is poor in mice, compared to rats and other species. Therefore, administration of a higher dose of the drug via the chow diet would not have necessarily resulted in significantly higher plasma concentrations. It seems possible that higher plasma concentrations of rivaroxaban might have translated not only to anti-inflammatory and lesion stabilizing effects, as observed in the present study, but also to a significant reduction of lesion progression. The rationale of using the present animal model was that long-term exposure to the drug in the genetically altered hyperlipidemic mice enables not only evaluation of lesion progression, but also, more relevant, of plaque composition. It also allows an, at least indirect comparison, between direct Factor Xa inhibition and direct thrombin inhibition, as evaluated by us in a previous study [8].

In our study, long-term administration of rivaroxaban (5 mg/kg/d) significantly decreased mRNA expression of Egr-1, IL-6, TNF-*α*, and MCP-1 in mouse aortic tissue, clearly demonstrating anti-inflammatory properties of Factor Xa inhibition. Mice, deficient in MCP-1 or its receptor CCR2, had significantly reduced atherosclerotic lesions, suggesting that MCP-1/CCR2 interaction has a role in monocyte recruitment in atherosclerosis [39, 40]. Although monocyte recruitment is an important proinflammatory event in atherogenesis, numbers of macrophages, as assessed by immunohistochemistry, were not reduced by rivaroxaban treatment in the present study. Further studies are needed to evaluate whether Factor Xa inhibition reduces activity of macrophages rather than the actual number of inflammatory cells in advanced atherosclerotic lesions.

## 5. Conclusion

In conclusion, our study demonstrates that chronic administration of rivaroxaban can exert anti-inflammatory effects, stabilizing lesions in a model of advanced atherosclerotic disease. Direct Factor Xa inhibitors might emerge as potential therapeutic tool in patients with established atherosclerotic disease.

## Figures and Tables

**Table 1:** Weight, rivaroxaban plasma concentrations, and lipids levels.

	Control	Low	High	*P*
Weight (g)	30.5 ± 3.4	29.4 ± 4.1	27.3 ± 3.5	*P < .01
Rivaroxaban concentration (ng/mL)	<2.0	6.5 ± 1.5	24.2 ± 9.3	*P < .01
Total cholesterol (mg/dL)	208.6 ± 68.0	218.8 ± 67.1	231.9 ± 55.0	ns
LDL cholesterol (mg/dL)	194.6 ± 68.5	203.4 ± 63.2	219.0 ± 51.9	ns
HDL cholesterol (mg/dL)	6.0 ± 2.1	4.9 ± 2.6	5.4 ± 2.9	ns
Triglycerides (mg/dL)	40.0 ± 16.8	52.0 ± 15.0	36.7 ± 12.0	ns

*high concentration versus control group.

**Table 2:** Morphologic lesion analysis A. Brachiocephalica.

	Control	Low	High	*P*
	(*n* = 19)	(*n* = 20)	(*n* = 20)	
Thin fibrous cap	16/19	17/20	17/20	ns
Large necrotic core	11/19	13/20	11/20	ns
Medial erosion	15/19	12/20	9/20	*P < .05
Calcification	12/19	13/20	12/20	ns
Cholesterol crystals	19/19	19/20	20/20	ns
Lateral xanthoma	7/19	3/20	1/20	*P < .05
Hemorrhage	11/19	7/20	6/20	ns

*high concentration versus control group.

## References

[1] Perzborn E, Strassburger J, Wilmen A (2005). In vitro and in vivo studies of the novel antithrombotic agent BAY 59-7939—an oral, direct Factor Xa inhibitor. *Journal of Thrombosis and Haemostasis*.

[2] Gensch C, Hoppe U, Böhm M, Laufs U (2011). Late-breaking clinical trials presented at the American Heart Association Congress in Chicago 2010. *Clinical Research in Cardiology*.

[3] The EINSTEIN Investigators (2010). Oral rivaroxaban for symptomatic venous thromboembolism. *New England Journal of Medicine*.

[4] Mega JL, Braunwald E, Mohanavelu S (2009). Rivaroxaban versus placebo in patients with acute coronary syndromes (ATLAS ACS-TIMI 46): a randomised, double-blind, phase II trial. *The Lancet*.

[5] Libby P (2001). Current concepts of the pathogenesis of the acute coronary syndromes. *Circulation*.

[6] Hansson GK (2005). Mechanisms of disease: inflammation, atherosclerosis, and coronary artery disease. *New England Journal of Medicine*.

[7] Harker LA, Hanson SR, Runge MS (1995). Thrombin hypothesis of thrombus generation and vascular lesion formation. *American Journal of Cardiology*.

[8] Bea F, Kreuzer J, Preusch M (2006). Melagatran reduces advanced atherosclerotic lesion size and may promote plaque stability in apolipoprotein E-deficient mice. *Arteriosclerosis, Thrombosis, and Vascular Biology*.

[9] Pfaffl MW (2001). A new mathematical model for relative quantification in real-time RT-PCR. *Nucleic acids research*.

[10] Davie EW, Fujikawa K, Kisiel W (1991). The coagulation cascade: Initiation, maintenance, and regulation. *Biochemistry*.

[11] Rao LVM, Pendurthi UR (2005). Tissue factor-factor VIIa signaling. *Arteriosclerosis, Thrombosis, and Vascular Biology*.

[12] Napoli C, De Nigris F, Wallace JL (2004). Evidence that protease activated receptor 2 expression is enhanced in human coronary atherosclerotic lesions. *Journal of Clinical Pathology*.

[13] Borensztajn K, Stiekema J, Nijmeijer S, Reitsma PH, Peppelenbosch MP, Spek CA (2008). Factor Xa stimulates proinflammatory and profibrotic responses in fibroblasts via protease-activated receptor-2 activation. *American Journal of Pathology*.

[14] Damiano BP, Cheung WM, Santulli RJ (1999). Cardiovascular responses mediated by protease-activated receptor-2 (PAR- 2) and thrombin receptor (PAR-1) are distinguished in mice deficient in PAR-2 or PAR-1. *Journal of Pharmacology and Experimental Therapeutics*.

[15] Grandaliano G, Pontrelli P, Cerullo G (2003). Protease-activated receptor-2 expression in IgA nephropathy: a potential role in the pathogenesis of interstitial fibrosis. *Journal of the American Society of Nephrology*.

[16] Masamune A, Kikuta K, Satoh M, Suzuki N, Shimosegawa T (2005). Protease-activated receptor-2-mediated proliferation and collagen production of rat pancreatic stellate cells. *Journal of Pharmacology and Experimental Therapeutics*.

[17] Cirino G, Cicala C, Bucci M (1997). Factor Xa as an interface between coagulation and inflammation: Molecular mimicry of factor Xa association with effector cell protease receptor-1 induces acute inflammation in vivo. *Journal of Clinical Investigation*.

[18] Senden NHM, Jeunhomme TMAA, Heemskerk JWM (1998). Factor Xa induces cytokine production and expression of adhesion molecules by human umbilical vein endothelial cells. *Journal of Immunology*.

[19] Papapetropoulos A, Piccardoni P, Cirino G (1998). Hypotension and inflammatory cytokine gene expression triggered by factor Xa-nitric oxide signaling. *Proceedings of the National Academy of Sciences of the United States of America*.

[20] Ruf W, Dorfleutner A, Riewald M (2003). Specificity of coagulation factor signaling. *Journal of Thrombosis and Haemostasis*.

[21] Hezi-Yamit A, Wong PW, Bien-Ly N (2005). Synergistic induction of tissue factor by coagulation factor Xa and TNF: evidence for involvement of negative regulatory signaling cascades. *Proceedings of the National Academy of Sciences of the United States of America*.

[22] Daubie V, Cauwenberghs S, Senden NHM (2006). Factor Xa and thrombin evoke additive calcium and proinflammatory responses in endothelial cells subjected to coagulation. *Biochimica et Biophysica Acta - Molecular Cell Research*.

[23] McLean K, Schirm S, Johns A, Morser J, Light DR (2001). FXa-induced responses in vascular wall cells are PAR-mediated and inhibited by ZK-807834. *Thrombosis Research*.

[24] Johnson K, Aarden L, Choi Y, De Groot E, Creasey A (1996). The proinflammatory cytokine response to coagulation and endotoxin in whole blood. *Blood*.

[25] Wakefield TW, Strieter RM, Wilke CA (1995). Venous thrombosis-associated inflammation and attenuation with neutralizing antibodies to cytokines and adhesion molecules. *Arteriosclerosis, Thrombosis, and Vascular Biology*.

[26] Dörffler-Melly J, Schwarte LA, Ince C, Levi M (2000). Mouse models of focal arterial and venous thrombosis. *Basic Research in Cardiology*.

[27] Turpie AGG (2007). Oral, direct factor Xa inhibitors in development for the prevention and treatment of thromboembolic diseases. *Arteriosclerosis, Thrombosis, and Vascular Biology*.

[28] Nicholson AC, Nachman RL, Altieri DC (1996). Effector cell protease receptor-1 is a vascular receptor for coagulation factor Xa. *Journal of Biological Chemistry*.

[29] Ko FN, Yang YC, Huang SC, Ou JT (1996). Coagulation factor Xa stimulates platelet-derived growth factor release and mitogenesis in cultured vascular smooth muscle cells of rat. *Journal of Clinical Investigation*.

[30] Gasic GP, Arenas CP, Gasic TB, Gasic GJ (1992). Coagulation factors X, Xa, and protein S as potent mitogens of cultured aortic smooth muscle cells. *Proceedings of the National Academy of Sciences of the United States of America*.

[31] Kaiser B (2003). DX-9065a, a direct inhibitor of factor Xa. *Cardiovascular Drug Reviews*.

[32] Ragosta M, Gimple LW, Gertz SD (1994). Specific factor Xa inhibition reduces restenosis after balloon angioplasty of atherosclerotic femoral arteries in rabbits. *Circulation*.

[33] Jang Y, Guzman LA, Lincoff AM (1995). Influence of blockade at specific levels of the coagulation cascade on restenosis in a rabbit atherosclerotic femoral artery injury model. *Circulation*.

[34] Lyle EM, Fujita T, Conner MW, Connolly TM, Vlasuk GP, Lynch JL (1995). Effect of inhibitors of factor X(a) or platelet adhesion, heparin, and aspirin on platelet deposition in an atherosclerotic rabbit model of angioplasty injury. *Journal of Pharmacological and Toxicological Methods*.

[35] Kopp CW, Hölzenbein T, Steiner S (2004). Inhibition of restenosis by tissue factor pathway inhibitor: in vivo and in vitro evidence for suppressed monocyte chemoattraction and reduced gelatinolytic activity. *Blood*.

[36] Tomasek JJ, Gabbiani G, Hinz B, Chaponnier C, Brown RA (2002). Myofibroblasts and mechano: Regulation of connective tissue remodelling. *Nature Reviews Molecular Cell Biology*.

[37] Xiong J, Zhu Z, Liu J, Wang Y, Li Z (2005). Role of protease activated receptor-2 expression in renal interstitial fibrosis model in mice. *Journal of Huazhong University of Science and Technology. Medical Sciences*.

[38] Bea F, Blessing E, Shelley MI, Shultz JM, Rosenfeld ME (2003). Simvastatin inhibits expression of tissue factor in advanced atherosclerotic lesions of apolipoprotein E deficient mice independently of lipid lowering: potential role of simvastatin-mediated inhibition of Egr-1 expression and activation. *Atherosclerosis*.

[39] Gu L, Okada Y, Clinton SK (1998). Absence of monocyte chemoattractant protein-1 reduces atherosclerosis in low density lipoprotein receptor-deficient mice. *Molecular Cell*.

[40] Boring L, Gosling J, Cleary M, Charo IF (1998). Decreased lesion formation in CCR2(-/-) mice reveals a role for chemokines in the initiation of atherosclerosis. *Nature*.

